# X-ray-Induced Scintillation Properties of Nd-Doped Bi_4_Si_3_O_12_ Crystals in Visible and Near-Infrared Regions

**DOI:** 10.3390/ma15248784

**Published:** 2022-12-08

**Authors:** Kensei Ichiba, Kai Okazaki, Yuma Takebuchi, Takumi Kato, Daisuke Nakauchi, Noriaki Kawaguchi, Takayuki Yanagida

**Affiliations:** Division of Materials Science, Nara Institute of Science and Technology (NAIST), Nara 630-0192, Japan

**Keywords:** scintillator, radiation detection, near-infrared luminescence, Bi_4_Si_3_O_12_

## Abstract

Undoped, 0.5, 1.0, and 2.0% Nd-doped Bi_4_Si_3_O_12_ (BSO) crystals were synthesized by the floating zone method. Regarding photoluminescence (PL) properties, all samples had emission peaks due to the 6p–6s transitions of Bi^3+^ ions. In addition, the Nd-doped samples had emission peaks due to the 4f–4f transitions of Nd^3+^ ions as well. The PL quantum yield of the 0.5, 1.0, and 2.0% Nd-doped samples in the near-infrared range were 67.9, 73.0, and 56.6%, respectively. Regarding X-ray-induced scintillation properties, all samples showed emission properties similar to PL. Afterglow levels at 20 ms after X-ray irradiation of the undoped, 0.5, 1.0, and 2.0% Nd-doped samples were 192.3, 205.9, 228.2, and 315.4 ppm, respectively. Dose rate response functions had good linearity from 0.006 to 60 Gy/h for the 1.0% Nd-doped BSO sample and from 0.03 to 60 Gy/h for the other samples.

## 1. Introduction

Scintillators are a kind of phosphor material that can immediately convert absorbed ionizing radiation energy to low-energy photons. As such, scintillators have played a key role in various fields, including medical imaging [1], security [2], astrophysics [3], and high-energy physics [4]. Generally, the required properties for scintillators are high effective atomic number (*Z*_eff_), high density, high radiation hardness, low afterglow level, and high chemical stability when the target ionizing radiations are high-energy photons (X- and γ-rays). Up to now, various scintillators such as Bi_4_Ge_3_O_12_ (BGO), Tl-doped CsI, Ce-doped Gd_3_Al_2_Ga_3_O_12_, Ce-doped Y_3_Al_5_O_12_, and CeBr_3_ have been developed to satisfy these criteria as much as possible [5,6,7,8,9]. In addition, such developments have mainly focused on materials emitting photons from the ultraviolet (UV) to visible (Vis) spectra because the wavelength sensitivity of photomultiplier tube (PMT), which is the most common photodetector in radiation detectors, is the UV–Vis range [10,11,12,13,14,15,16].

Recently, InGaAs-based photodetectors with wavelength sensitivity in the near-infrared (NIR) region have been widely commercialized, and scintillators emitting NIR photons (NIR scintillators) have attracted attention. NIR scintillators are considered to have various applications, such as dose monitoring in high-dose environments and in medical therapy. In high-dose environments, such as inside of nuclear reactors, Cherenkov light occurs near some components of radiation detectors [17]. Because the wavelength of Cherenkov light is widely distributed over the UV–Vis range, it is difficult for typical scintillators to distinguish UV–Vis signal photons emitted from scintillators with noise from Cherenkov light. However, NIR scintillators can easily discriminate between these two kinds of light. If one uses NIR scintillators and photodetectors only sensitive to NIR photons, Cherenkov light cannot be detected at all. In terms of medical therapy, because NIR photons have the advantage of high biological transparency, they have been suggested for use in drug delivery systems [18,19]. The proposed systems would maximize the effects of delivered drugs as well as allow drugs inside the body to be controlled and monitored [20,21]. Using a drug delivery system can be less damaging to patients’ bodies than normal radiation and chemotherapies. Despite various merits and the wide range of potential applications, there have been fewer investigations for NIR scintillators than for typical scintillators because it is difficult to measure the scintillation photons in the NIR region. Recently, we have developed measurement systems for NIR scintillators, and some NIR scintillators such as BGO, LaVO_4_, GdVO_4_, and YAlO_3_ [22,23,24,25] have been introduced.

Following the above investigations of NIR scintillators, in this study, we focused on Bi_4_Si_3_O_12_ (BSO), which has a similar composition to BGO. BSO has been investigated as a UV–Vis scintillator for a long time because it has high chemical stability, low afterglow level, high *Z*_eff_ (77.3), and high density (6.80 g/cm^3^) [26]. Even though the density of BSO is inferior to that of BGO, the *Z*_eff_ of BSO is higher than that of BGO. In addition, BSO exhibits about 10–100 times higher radiation hardness than BGO [27]. This indicates that BSO is the superior host over BGO for the application of a monitoring device in a high-dose environment. Furthermore, because SiO_2_ raw powder is less expensive than GeO_2_ raw powder, BSO can be produced at a lower cost than BGO. However, an investigation of the scintillation properties of BSO as an NIR scintillator has never been conducted. As a luminescence center, we focused on the Nd^3+^ ion. The Nd^3+^ ion can emit NIR photons, and the main emission peak occurs at 1060 nm, which is located in the second biological window [28,29]. Therefore, the Nd^3+^ ion is a promising luminescence center for an NIR scintillator.

## 2. Materials and Methods

Undoped and Nd-doped BSO crystals were synthesized by the floating zone (FZ) method, and Nd concentrations of 0.5, 1.0, and 2.0% were chosen. The raw powders were Bi_2_O_3_ (99.99%, Rare Metallic, Tokyo, Japan), SiO_2_ (99.99%, Rare Metallic, Tokyo, Japan), and Nd_2_O_3_ (99.99%, Rare Metallic, Tokyo, Japan). After each raw powder was homogeneously mixed with an agate mortar and pestle, the mixture was shaped into a cylindrical rod by applying hydrostatic pressure. The shaped rod was sintered at 800 °C for 8 h in air. Then, the sintered rod was used to conduct crystal growth by using an FZ furnace (Canon Machinery Inc., Shiga, Japan, FZD0192) with a pull-down rate of 5 mm/h and a rotation speed of 10 rpm. After the crystal growth phase, a small piece was taken from the now-crystalline rod. The surface of the piece was polished by a mechanical polishing machine (Buehler, Lake Bluff, IL, USA, MetaServ 250). For the confirmation of the existence of the crystalline phase, the powder X-ray diffraction (PXRD) patterns were measured in the 10–90° range with a diffractometer (Rigaku, Tokyo, Japan, MiniFlex600). For this measurement, the remaining part of the crystalline rod was crushed and used.

The PL excitation and emission mapping and PL quantum yield (*QY*) were evaluated with a Quantaurus-QY Plus machine (Hamamatsu, Shizuoka, Japan, C13534), which was equipped with a Xe lamp as an excitation light source. In addition, a Si linear image sensor was used for measurements in the emission range of 250–950 nm, and an InGaAs linear image sensor for 950–1650 nm. The absolute PL *QY* was calculated by the formula of *QY* = *N*_emit_/*N*_absorb_. Here, *N*_emit_ and *N*_absorb_ indicate the number of emitted and absorbed photons, respectively. The PL decay time profiles were measured with a Quantaurus-τ (Hamamatsu, Shizuoka, Japan, C11367).

Regarding scintillation properties, the X-ray-induced scintillation spectra were assessed with our original setup [30]. The X-ray source was an X-ray generator (Spellman, Hauppauge, USA, XRB80N100/CB). The scintillation photons were transported through an optical fiber to a monochromator (Andor, Belfast, England, Shamrock 163) and a Si-based line camera (Andor, Belfast, England, DU-420-BU2) for measurements in the range of 200–700 nm or an InGaAs-based line camera (Andor, Belfast, UK, DU492A) for measurements in the range of 700–1600 nm. First, background signals mainly due to thermal noise were evaluated without the sample, and the background spectrum was subtracted from the measured spectra with the sample. In measurements, X-ray irradiation for 10 s was repeated 6 times, and the integrated luminescence intensity in each wavelength was read. The tube voltage and current of the X-ray generator were set to 80 kV and 1.2 mA, respectively, for the measurement in the 200–700 nm range and to 40 kV and 1.2 mA, respectively, for the measurement for the 700–1600 nm range. X-ray-induced scintillation decay time profiles and afterglow profiles were investigated with an afterglow characterization system [31]. In this system, a PMT (Hamamatus, Shizuoka, Japan, R7400P-06) was employed for monitoring at 160–700 nm, and a different PMT (Hamamatus, Shizuoka, Japan, H7421–50) was used for monitoring at 380–900 nm.

To investigate the performance as a NIR scintillation detector, the dose-rate–response function was evaluated with our original setup [32]. Utilizing an InGaAs PIN photodiode (Hamamatsu, Shizuoka, Japan, G12180–250A) through an optical fiber (Thorlabs, Newton, NJ, USA, FP600ER,) with picoammeter (Keysight, Santa Rosa, CA, USA, B2985A), the emission intensity in the NIR range was evaluated under various dose rates of X-rays.

## 3. Results and Discussion

After the crystal growth phase, crystalline rods typically 4 mmϕ × 20 mm in length were obtained. For all measurements except for PXRD, the dimensions of all samples retrieved from crystalline rods were approximately 4 × 4 × 1 mm. Some of the remaining parts of the sampled crystalline rods were crushed to powder form to measure the PXRD.

**Figure 1:**
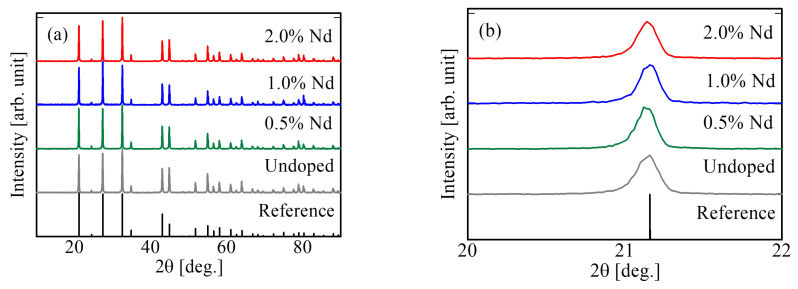
PXRD patterns of undoped, 0.5, 1.0, and 2.0% Nd-doped BSO crystals and reference patterns (COD 9012894) in the range of (**a**) 10–90° and (**b**) 20–22°.

Figure 1a shows the PXRD patterns of the synthesized undoped, 0.5, 1.0, and 2.0% Nd-doped BSO crystals and the reference pattern of BSO in the Crystallography Open Database No. 9012894 (COD 9012894). The diffraction peaks of the synthesized samples were observed at the same positions as the reference patterns. Therefore, the synthesized samples had the single-phase structures of BSO. The enlarged PXRD patterns in the range of 20–22° are shown in Figure 1b. Although Nd^3+^ ions occupy the Bi^3+^ sites in the host lattice [33], no peak shift was confirmed because Nd^3+^ and Bi^3+^ ions in BSO have almost the same ionic radius (Nd^3+^: 1.11 Å and Bi^3+^: 1.17 Å).

**Figure 2:**
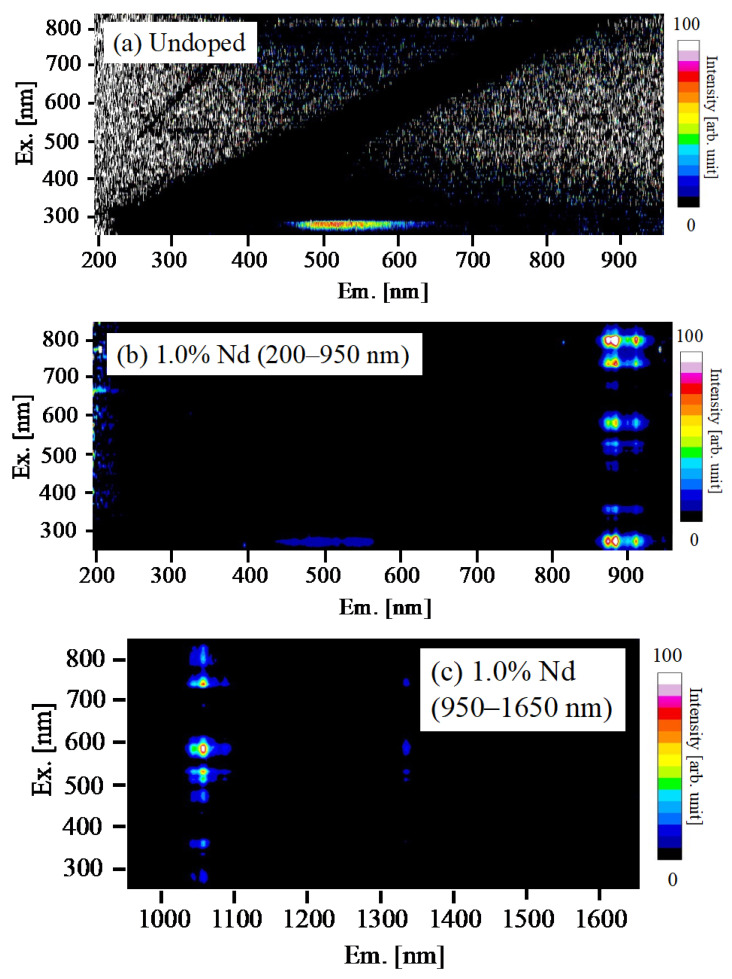
PL excitation and emission mapping of (**a**) undoped, (**b**) 1.0% Nd-doped BSO crystal in the emission range of 200–950 nm, and (**c**) 1.0% Nd-doped BSO crystal in the range of 950–1650 nm. The horizontal and vertical axes show the emission and excitation wavelengths, respectively.

Figure 2 shows PL excitation and emission mapping of undoped, 1.0% Nd-doped BSO crystal in the emission range of 200–950 nm, and 1.0% Nd-doped BSO crystal in the emission range of 950–1650 nm. Both the samples had an emission band at around 500 nm under the excitation wavelength of 280 nm. The emission band was derived from the 6p–6s (^3^P_1_–^1^S_0_) transitions of Bi^3+^ ions [34]. Furthermore, the 1.0% Nd-doped sample had some emission peaks at 880, 1060, and 1340 nm under the excitation wavelength in the range of 250–800 nm within the emission band due to Bi^3+^ ions. The origin of emission peaks at 880, 1060, and 1340 nm were the 4f–4f (^4^F_3/2_–^4^I_11/2_ and ^4^F_3/2_–^4^I_13/2_, respectively) transitions of Nd^3+^ ions [35,36]. It was noted that the PL excitation and emission mapping of other Nd-doped samples also indicated the same tendencies as that of the 1.0% Nd-doped sample. The PL *QY* of the undoped, 0.5, 1.0, and 2.0% Nd-doped samples monitored at 400–700 nm were 2.4, 1.6, 1.1, and 0.7% under excitation at 280 nm, respectively. As the Nd concentration increased, the PL *QY* derived from the emission of Bi^3+^ ions decreased. In the range of 1000–1600 nm, the maximum PL *QY* of the 0.5, 1.0, and 2.0% Nd-doped samples were obtained under excitation at 580 nm, and the values were 67.9, 73.0, and 56.6%, respectively. The PL *QY*s of the Nd-doped samples surpassed that of the Nd-doped BGO crystal (the maximum value: 42.9%) [35]. The decrease in the PL *QY* in the 2.0% Nd-doped sample would be due to the concentration quenching.

**Figure 3:**
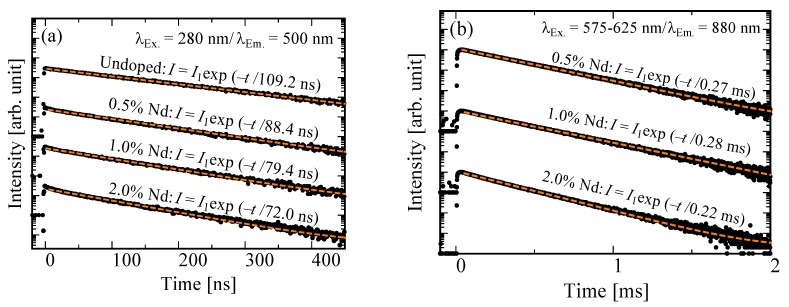
(**a**) PL decay time profiles of undoped, 0.5, 1.0, and 2.0% Nd-doped BSO crystals monitoring at 500 nm under 280 nm excitation; (**b**) those of 0.5, 1.0, and 2.0% Nd-doped BSO crystals monitoring at 880 nm under 575–625 nm excitation.

Figure 3 shows the PL decay time profiles of the undoped, 0.5, 1.0, and 2.0% Nd-doped BSO crystals. Each excitation/monitoring wavelength was 280/500 nm and 575–625/880 nm. All decay curves could be well-reproduced by a single exponential decay function excluding the instrumental response function (IRF). In Figure 3a, the obtained decay time constants of the undoped, 0.5, 1.0, and 2.0% Nd-doped samples are 109.2, 88.4, 79.4, and 72.0 ns, respectively. These values related to the 6p–6s transitions of Bi^3+^ ions [37]. In addition, the obtained decay time constants decreased with increasing concentration of Nd^3+^ ions. Because the emission band of Bi^3+^ ions overlapped the excitation of Nd^3+^ ions (shown in Figure 2), the decrease in decay time constants was caused by an energy transfer from Bi^3+^ ions to Nd^3+^ ions. The energy transfer from Bi^3+^ ion to Nd^3+^ ions would also be supported by the decrease in the PL *QY* derived from Bi^3+^ ions with increasing Nd concentration. In Figure 3b, the obtained decay time constants of the 0.5, 1.0, and 2.0% Nd-doped samples are 0.27, 0.28, and 0.22 ms, respectively. These values corresponded to the typical value due to the 4f–4f transitions of the Nd^3+^ ions [38]. In addition, we were unable to evaluate the PL decay time constants monitored at the 1060 nm due to the spectral sensitivity limitation of the PMT, but the decay time at 1060 nm would be in a similar range.

**Figure 4:**
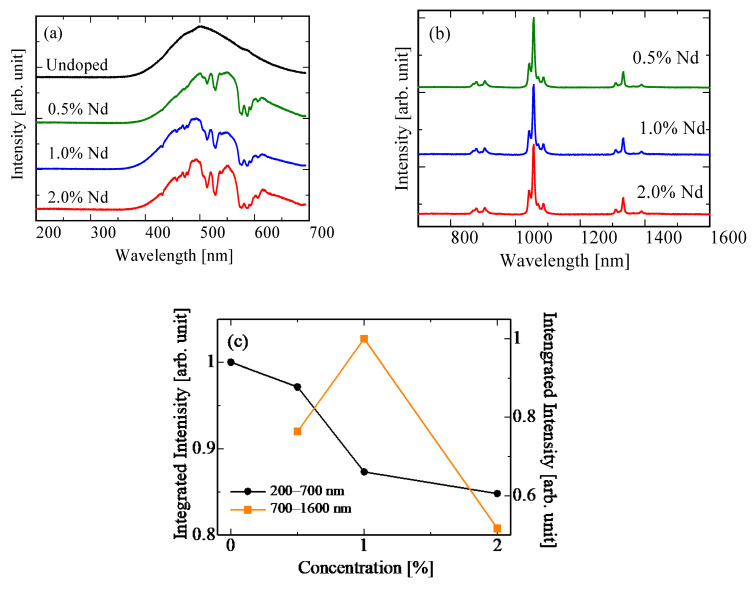
X-ray-induced scintillation spectra of (**a**) undoped, 0.5, 1.0, and 2.0% Nd-doped BSO crystals in the range of 200–700 nm and (**b**) 0.5, 1.0, and 2.0% Nd-doped BSO crystals in the range of 700–1600 nm. (**c**) The integrated intensities of undoped, 0.5, 1.0, and 2.0% Nd-doped BSO crystals in the range of 200–700 nm and that of 0.5, 1.0, and 2.0% Nd-doped BSO crystals in the range of 700–1600 nm after normalization by the maximum intensity.

Figure 4 shows the X-ray-induced scintillation spectra of the undoped, 0.5, 1.0, and 2.0% Nd-doped BSO crystals in the range of 200–700 nm and 700–1600 nm. In the range of 200–700 nm, the emission band was observed at 400–700 nm. This emission band was due to the 6p–6s (^3^P_1_–^1^S_0_) transitions of Bi^3+^ ions [39,40]. Regarding the Nd-doped samples, a decrease in emission intensities at some wavelengths (e.g., ~580 nm) was observed at the emission band of the BSO host in 400–700 nm. The wavelengths where the decrease was observed were consistent with the PL excitation peaks due to the 4f–4f transitions of the Nd^3+^ ions. Figure 4c shows the integrated intensities of undoped, 0.5, 1.0, and 2.0% Nd-doped BSO crystals in the range of 200–700 nm after normalization by the maximum intensity. Generally, intensities of X-ray-induced scintillation spectra are qualitative values, which are difficult to compare. However, because we used the samples with similar chemical compositions and sample sizes, intensities could be compared. The decrease in emission intensity grew more pronounced as Nd concentration increased. Therefore, this decrease was caused by absorption due to the 4f–4f transitions of the Nd^3+^ ions. The same phenomenon was shown in the scintillation spectra of Nd-doped BGO [35]. In the range of 700–1600 nm, the Nd-doped samples had some emission peaks at approximately 900, 1060, and 1340 nm, which were ascribed to the luminescence due to the 4f–4f (^4^F_3/2_–^4^I_9/2_, ^4^F_3/2_–^4^I_11/2_, and ^4^F_3/2_–^4^I_13/2_, respectively) transitions of Nd^3+^ ions [41]. In addition, Figure 4c shows the integrated intensities of 0.5, 1.0, and 2.0% Nd-doped BSO crystals in the range of 700–1600 nm after normalization by the maximum intensity. The integrated intensity in the range of 700–1600 nm was consistent with the trend in PL *QY* in the range of 1000–1600 nm.

**Figure 5:**
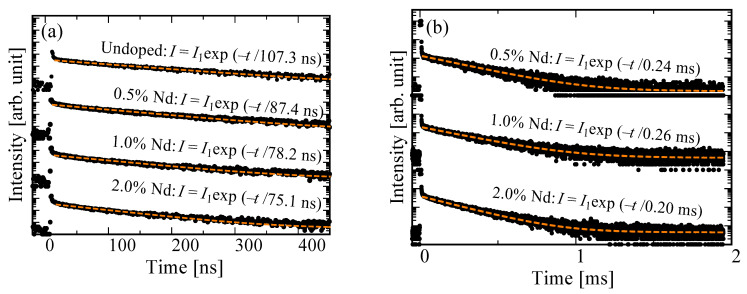
(**a**) X-ray-induced scintillation decay time profiles of undoped, 0.5, 1.0, and 2.0% Nd-doped BSO crystals in the monitoring range of 160–650 nm and (**b**) those of 0.5, 1.0, and 2.0% Nd-doped BSO crystals in the monitoring range of 380–900 nm.

Figure 5 shows the X-ray-induced scintillation decay time profiles of the undoped, 0.5, 1.0, and 2.0% Nd-doped BSO crystals by using PMTs, which had sensitivities in the range of 160–650 nm and 380–900 nm. All decay curves were approximated by a single exponential function excluding the IRF. In Figure 5a, the obtained decay time constants of the undoped, 0.5, 1.0, and 2.0% Nd-doped samples are 107.3, 87.4, 78.4, and 75.1 ns, respectively. These decay time constants were the 6p–6s transitions of Bi^3+^ ions [35], and their decrease with decreasing Nd concentration was due to the energy transfer from Bi^3+^ ions to Nd^3+^ ions. In Figure 5b, the obtained decay time constants of the 0.5, 1.0, and 2.0% Nd-doped samples were 0.24, 0.26, and 0.20 ms, respectively, values that were typical in the 4f–4f transitions of Nd^3+^ ions [42]. In addition, the decay time constants shown in Figure 5b were derived from the value that was mainly due to the emission at 900 nm of Nd^3+^ ions because the monitoring range was 380–900 nm. The decay time constants of the Nd-doped samples are quite long if used for the photon counting measurement. However, the Nd-doped samples can be applied to dose monitoring in high-dose environments, which requires an integration-type measurement. In this kind of measurement, the integration time is several seconds order.

**Figure 6:**
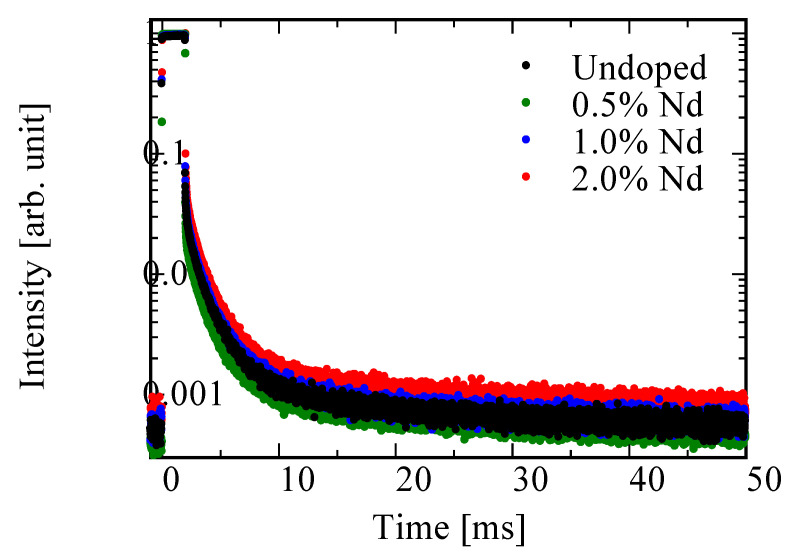
Afterglow profiles (X-ray-induced) of undoped, and 0.5, 1.0, and 2.0% Nd-doped BSO crystals.

Figure 6 shows the afterglow profiles (X-ray-induced) of the undoped, 0.5, 1.0, and 2.0% Nd-doped BSO crystals. The afterglow level (*Af*) was calculated by the following formula:

(1) $$Af=\frac{I_{20}-I_{\mathrm{BG}}}{I_{\mathrm{MAX}}-I_{\mathrm{BG}}}$$

where *I*_BG_ is the background signal before X-ray irradiation, *I*_20_ is the signal intensity after 20 ms cutting off the X-ray irradiation, and *I*_MAX_ is the signal intensity during X-ray irradiation. Using the above formula, the obtained values of the undoped, 0.5, 1.0, and 2.0% Nd-doped samples were 192.3, 205.9, 228.2, and 315.4 ppm, respectively. The *Af* increased as Nd concentration increased. Afterglow occurs when the carriers captured by the shallow trapping centers in the material are re-excited by room temperature and are transferred to luminescence centers. Therefore, the increase in *Af* would be caused by the increase in trap sites owing to doping Nd^3+^ ions. In addition, the *Af* of BSO was larger than that of BGO [35]. This fact would indicate that the number of shallow trapping centers was higher in BSO than in BGO.

**Figure 7:**
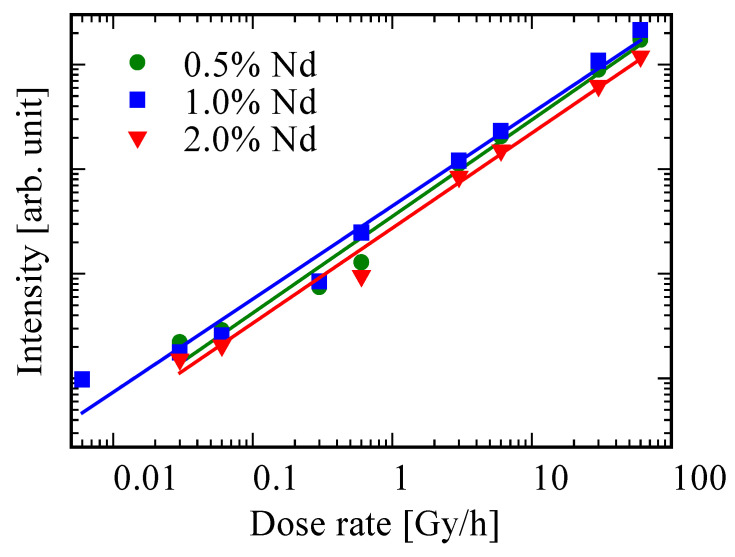
Dose-rate–response functions of 0.5, 1.0, and 2.0% Nd-doped BSO crystals.

Figure 7 shows the dose-rate–response functions of the 0.5, 1.0, and 2.0% Nd-doped BSO crystals to investigate the performance as a NIR scintillation detector. The linear relationship was observed from 0.03 to 60 Gy/h for the 0.5 and 2.0% Nd-doped samples and from 0.006 to 60 Gy/h for the 1.0% Nd-doped sample. Here, the determination coefficients of the 0.5, 1.0, and 2.0% Nd-doped samples were 0.983, 0.982, 0.989, respectively. These coefficients imply that the functions were well-approximated. The lowest detection limit of the 1.0% Nd-doped sample was lower than that of Nd-doped BGO (0.01 Gy/h) measured with the same setup [35].

## 4. Conclusions

Undoped, 0.5, 1.0, and 2.0% Nd-doped BSO crystals were synthesized by the FZ method. All the Nd-doped samples had emission due to the 6p–6s transitions of Bi^3+^ ions and the 4f–4f transitions of Nd^3+^ ions in their PL properties. In addition, the PL *QY* of the 1.0% BSO samples in near-infrared range were 73.0%, which is the highest value among the Nd-doped samples reported so far. In X-ray-induced scintillation properties, all Nd-doped samples shared similar emission properties with PL. The *Af* of the undoped, 0.5, 1.0, and 2.0% Nd-doped samples were 192.3, 205.9, 228.2, and 315.4 ppm, respectively. Dose-rate–response functions had good linearity from 0.006 to 60 Gy/h for the 1.0% Nd-doped BSO sample and from 0.03 to 60 Gy/h for other samples. The lower detection limit of the 1.0% Nd-doped BSO crystal was lower than that of Nd-doped BGO. In conclusion, Nd-doped BSO crystals are viable candidates for NIR scintillators.

## Data Availability

Data will be made available on request.

## References

[1] Van Eijk C.W.E. (2002). Inorganic scintillators in medical imaging. Phys. Med. Biol..

[2] Liu Q., Cheng Y., Yang Y., Peng Y., Li H., Xiong Y., Zhu T. (2020). Image reconstruction using multi-energy system matrices with a scintillator-based gamma camera for nuclear security applications. Appl. Radiat. Isot..

[3] Picozza P., Galper A.M., Castellini G., Adriani O., Altamura F., Ambriola M., Barbarino G.C., Basili A., Bazilevskaja G.A., Bencardino R. (2007). PAMELA—A payload for antimatter matter exploration and light-nuclei astrophysics. Astropart. Phys..

[4] Mao R., Zhang L., Zhu R.-Y. (2008). Optical and Scintillation Properties of Inorganic Scintillators in High Energy Physics. IEEE Trans. Nucl. Sci..

[5] Yanagida T., Fujimoto Y., Koshimizu M., Watanabe K., Sato H., Yagi H., Yanagitani T. (2014). Positive hysteresis of Ce-doped GAGG scintillator. Opt. Mater..

[6] Nagarkar V.V., Gupta T.K., Miller S.R., Klugerman Y., Squillante M.R., Entine G. (1998). Structured CsI(Tl) scintillators for X-ray imaging applications. IEEE Trans. Nucl. Sci..

[7] Moszyński M., Gresset C., Vacher J., Odru R. (1981). Timing properties of BGO scintillator. Nucl. Instrum. Methods Phys. Res..

[8] Moszyński M., Ludziejewski T., Wolski D., Klamra W., Norlin L.O. (1994). Properties of the YAG:Ce scintillator. Nucl. Instrum. Methods Phys. Res. Sect. A.

[9] Quarati F.G.A., Alekhin M.S., Krämer K.W., Dorenbos P. (2014). Co-doping of CeBr_3_ scintillator detectors for energy resolution enhancement. Nucl. Instrum. Methods Phys. Res. Sect. A.

[10] Ichiba K., Takebuchi Y., Kimura H., Kato T., Nakauchi D., Kawaguchi N., Yanagida T. (2021). Radiation-induced luminescence properties of Ce–doped Mg_2_SiO_4_ single crystals. J. Mater. Sci. Mater. Electron..

[11] Moszyński M., Wolski D., Ludziejewski T., Kapusta M., Lempicki A., Brecher C., Wiśniewski D., Wojtowicz A. (1997). Properties of the new LuAP:Ce scintillator. Nucl. Instrum. Methods Phys. Res. Sect. A.

[12] Ichiba K., Takebuchi Y., Kimura H., Kato T., Nakauchi D., Kawaguchi N., Yanagida T. (2022). Photoluminescence, scintillation, and dosimetric properties of Tb-doped Mg_2_SiO_4_ single crystals. J. Mater. Sci. Mater. Electron..

[13] Yanagida T. (2018). Inorganic scintillating materials and scintillation detectors. Proc. Jpn. Acad. Ser. B.

[14] Zhang Z., Guo X., Huang K., Sun X., Li X., Zeng H., Zhu X., Zhang Y., Xie R. (2022). Lead-free bright yellow emissive Rb_2_AgCl_3_ scintillators with nanosecond radioluminescence. J. Lumin..

[15] Tseremoglou S., Michail C., Valais I., Ninos K., Bakas A., Kandarakis I., Fountos G., Kalyvas N. (2022). Efficiency Properties of Cerium-Doped Lanthanum Chloride (LaCl_3_:Ce) Single Crystal Scintillator under Radiographic X-ray Excitation. Crystals.

[16] Abdalla A.M., Almalki S., Kawaguchi N., Yanagida T. (2022). Nanostructured scintillator developed in-house for radon detection. Radiat. Phys. Chem..

[17] Madden L., Archer J., Li E., Wilkinson D., Rosenfeld A. (2018). Temporal separation of Cerenkov radiation and scintillation using artificial neural networks in Clinical LINACs. Phys. Medica.

[18] Zhu S., Yung B.C., Chandra S., Niu G., Antaris A.L., Chen X. (2018). Near-Infrared-II (NIR-II) Bioimaging via Off-Peak NIR-I Fluorescence Emission. Theranostics.

[19] Huang Y., Qiu F., Chen R., Yan D., Zhu X. (2020). Fluorescence resonance energy transfer-based drug delivery systems for enhanced photodynamic therapy. J. Mater. Chem. B.

[20] You J., Zhang R., Zhang G., Zhong M., Liu Y., Van Pelt C.S., Liang D., Wei W., Sood A.K., Li C. (2012). Photothermal-chemotherapy with doxorubicin-loaded hollow gold nanospheres: A platform for near-infrared light-trigged drug release. J. Control. Release.

[21] Gupta B.P., Thakur N., Jain N.P., Banweer J., Jain S. (2010). Osmotically Controlled Drug Delivery System with Associated Drugs. J. Pharm. Pharm. Sci..

[22] Okazaki K., Fukushima H., Nakauchi D., Okada G., Onoda D., Kato T., Kawaguchi N., Yanagida T. (2022). Investigation of Er:Bi_4_Ge_3_O_12_ single crystals emitting near-infrared luminescence for scintillation detectors. J. Alloys Compd..

[23] Akatsuka M., Kimura H., Onoda D., Shiratori D., Nakauchi D., Kato T., Kawaguchi N., Yanagida T. (2021). X-ray-induced Luminescence Properties of Nd-doped GdVO_4_. Sens. Mater..

[24] Akatsuka M., Nakauchi D., Kato T., Kawaguchi N., Yanagida T. (2020). Optical and Scintillation Properties of YAlO_3_ Doped with Rare-Earths Emitting Near-infrared Photons. Sens. Mater..

[25] Akatsuka M., Nakauchi D., Kato T., Kawaguchi N., Yanagida T. (2022). Characterization of Nd: LaVO_4_ single-crystal scintillator emitting near-infrared photons. Jpn. J. Appl. Phys..

[26] Lima A.F., Souza S.O., Lalić M.V. (2009). Electronic structure and optical absorption of the Bi_4_Ge_3_O_12_ and the Bi_4_Si_3_O_12_ scintillators in ultraviolet region: An ab initio study. J. Appl. Phys..

[27] Ishii M., Harada K., Hirose Y., Senguttuvan N., Kobayashi M., Yamaga I., Ueno H., Miwa K., Shiji F., Yiting F. (2002). Development of BSO (Bi_4_Si_3_O_12_) crystal for radiation detector. Opt. Mater..

[28] Dahal D., Ray P., Pan D. (2021). Unlocking the power of optical imaging in the second biological window: Structuring near-infrared II materials from organic molecules to nanoparticles. WIREs Nanomed. Nanobiotechnology.

[29] Tanaka J.T., Moscardini S.B., do Nascimento Melo W.E., Brunckova H., Nassar E.J., Rocha L.A. (2021). NIR Luminescence Enhancement of YVO_4_:Nd Phosphor for Biological Application. J. Fluoresc..

[30] Yanagida T., Kamada K., Fujimoto Y., Yagi H., Yanagitani T. (2013). Comparative study of ceramic and single crystal Ce:GAGG scintillator. Opt. Mater..

[31] Yanagida T., Fujimoto Y., Ito T., Uchiyama K., Mori K. (2014). Development of X-ray-induced afterglow characterization system. Appl. Phys. Express.

[32] Fukushima H., Akatsuka M., Kimura H., Onoda D., Shiratori D., Nakauchi D., Kato T., Kawaguchi N., Yanagida T. (2021). Optical and Scintillation Properties of Nd-doped Strontium Yttrate Single Crystals. Sens. Mater..

[33] Chen F., Ju M., Kuang X., Yeung Y. (2018). Insights into the Microstructure and Transition Mechanism for Nd^3+^-Doped Bi_4_Si_3_O_12_: A Promising Near-Infrared Laser Material. Inorg. Chem..

[34] Jiang H., Wang X., Hao G., Wang L. (2013). Molten salt synthesis and luminescence properties of Bi_4_Si_3_O_12_ powders. J. Mater. Sci. Mater. Electron..

[35] Okazaki K., Onoda D., Fukushima H., Nakauchi D., Kato T., Kawaguchi N., Yanagida T. (2021). Characterization of scintillation properties of Nd-doped Bi_4_Ge_3_O_12_ single crystals with near-infrared luminescence. J. Mater. Sci. Mater. Electron..

[36] Payziyev S., Sherniyozov A., Bakhramov S., Zikrillayev K., Khalikov G., Makhmudov K., Ismailov M., Payziyeva D. (2021). Luminescence sensitization properties of Ce: Nd: YAG materials for solar pumped lasers. Opt. Commun..

[37] Kang F., Peng M., Zhang Q., Qiu J. (2014). Abnormal Anti-Quenching and Controllable Multi-Transitions of Bi^3+^ Luminescence by Temperature in a Yellow-Emitting LuVO_4_: Bi^3+^ Phosphor for UV-Converted White LEDs. Chem.-A Eur. J..

[38] Hreniak D., Fedyk R., Bednarkiewicz A., Stręk W., Łojkowski W. (2007). Luminescence properties of Nd:YAG nanoceramics prepared by low temperature high pressure sintering method. Opt. Mater..

[39] Zhu X., Xie J., Lin D., Guo Z., Xu J., Shi Y., Lei F., Wang Y. (2014). Synthesis of BSO (Bi_4_Si_3_O_12_) scintillation thin film by sol–gel method. J. Alloys Compd..

[40] Hua J., Kim H.J., Rooh G., Park H., Kim S., Cheon J. (2011). Czochralski growth and scintillation properties of Bi_4_Si_3_O_12_ (BSO) single crystal. Nucl. Instrum. Methods Phys. Res. Sect. A.

[41] Santos H.D.A., Novais S.M.V., Jacinto C. (2018). Optimizing the Nd:YF_3_ phosphor by impurities control in the synthesis procedure. J. Lumin..

[42] Das S., Som S., Yang C., Lu C., Chen Y., Shy H. (2016). Synthesis and characterization of high concentration Nd^3+^ doped YAG nanopowders for laser applications. Proceedings of the 5th International Conference on Mechanical Engineering, Materials and Energy (5th ICMEME2016).

